# Induction of senescence like macrophages by hepatocellular carcinoma cells in vitro

**DOI:** 10.1007/s12672-026-05348-8

**Published:** 2026-06-01

**Authors:** Zhining Chen, Jing Huang, Jiabao Liang, Tong Wen, Xiaoling Luo

**Affiliations:** 1 Department of Immunology, School of Basic Medical Sciences, Guangxi Medical University, No 22, Shuang Yong Road, Nanning, 530021 China; 2 Key Laboratory of Early Prevention and Treatment for Regional High Frequency Tumor (Guangxi Medical University), Ministry of Education/Guangxi Key Laboratory of Early Prevention and Treatment for Regional High Frequency Tumor, Nanning, 530021 China; 3 Department of pathology, Guangxi Medical University Cancer Hospital, Nanning, 530021 China; 4 Department of Respiratory Oncology, Guangxi Medical University Cancer Hospital, Nanning, 530021 China; 5 Department of radiotherapy, Guangxi Medical University Cancer Hospital, Nanning, 530021 China

**Keywords:** Macrophages, Senescence-like, Hepatocellular carcinoma

## Abstract

Cellular senescence has been regarded as a tumor-suppressive mechanism when occurring in cancer cells. However, when senescence occurs in immune cells, where it can exert opposing effects. Macrophages, one of crucial component of the immune cell have been shown to undergo senescence in mouse models of glioma and lung cancer. Yet, evidence for macrophage senescence in hepatocellular carcinoma (HCC) remains limited. To investigate the senescent state of macrophage in HCC we established an in vitro co-culture model of murine hepatocellular carcinoma cell line Hepa1-6 and macrophages-like transformed murine cell line RAW264.7. SA-β-Gal staining, real-time quantitative PCR, bulk RNA-seq, immunofluorescence were employed to demonstrate macrophage senescence which co-culture with hepatocellular carcinoma. Real-time qPCR, phagocytosis assays, flow cytometry, and transwell invasion experiments revealed the function of macrophages. RAW 264.7 cells exhibited morphological changes consistent with senescence-like change, along with elevated staining of the senescence-associated marker SA-β-Gal and higher proportion of G1 phase in Cell cycle analysis. Co-cultured RAW264.7 cells exhibited upregulated expression of senescence-related genes while downregulating proliferation and cell cycle-related genes. Additionally, senescent macrophages displayed impaired function and adopted an M2-like phenotype. In summary, our findings indicate that macrophages co-cultured with hepatocellular carcinoma cells acquire senescence-like phenotype and the function has also changed.

**Graphical Abstract:**

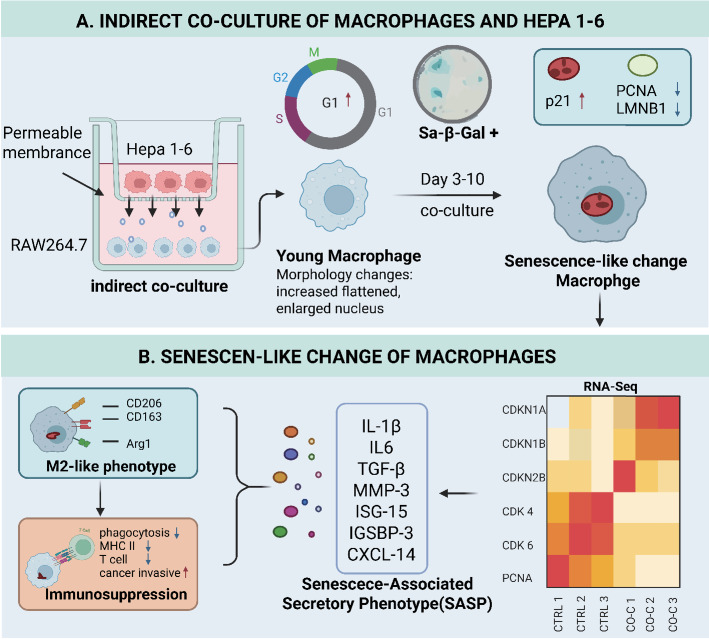

**Supplementary Information:**

The online version contains supplementary material available at 10.1007/s12672-026-05348-8.

## Introduction

Cellular senescence, marked by changes in gene activity and cessation of cell division [1–3], which plays a significant role in various diseases, including tumor. Since senescence can restrict cell proliferation, thereby inhibiting the unlimited growth of highly proliferative cells such as tumor cells [4], and senescence-associated secretory phenotype (SASP) factors—such as tumor necrosis factor TNF-α, IL-1α, IL-1β, IL-6, CXCL1, and interferon IFN-γ [5, 6]—mediate intercellular communication to exert tumor-suppressive effects. Many studies have focused on tumor cells senescence, however, as research progresses, it has been revealed that senescence can also exert multiple pro-tumorigenic effects [7]. There are three main factors. Initially, senescence involves more than just the halting of the cell cycle; it also includes a transformation in the gene expression profiles during the senescent state. This alteration, for instance, leads to the enhancement of genes related to the senescent phenotype, resulting in the secretion of cytokines through autocrine and paracrine signaling that alters the tumor microenvironment (TME), some of these factors can promote tumor initiation, invasion, and metastasis. Additionally, tumor cells can exploit the genetic or phenotypic changes when senescence to escape from the function of immune surveillance and clearance by immune cells. Furthermore, senescence does not occur only in tumor cells, when other cell types for example immune cells in TEM undergo senescence, they can play different roles [8].

Macrophages are a crucial component of the immune system. When macrophages that infiltrate tumor tissues or accumulate in the microenvironment of solid tumors are defined as tumor-associated macrophages (TAMs) [9]. TAMs were once considered as anti-tumor effector cells with capacity of killing tumor or inducing an immune response to eliminate tumors [10]. However, a significant proportion of TAMs exhibit low expression of MHC class II molecules, upregulate the expression of immune check point such as PD-L1, Tim-3 and secret cytokines like VEGF, TGF-β, and MMP3 to promote tumor growth and metastasis [6, 11, 12]. In some mouse models, these pro-tumorigenic TAMs display features of senescence. For instance, in an immunocompetent glioma mouse model, macrophage senescence was found to play a critical role in tumor initiation and steady growth [13]; Additionally, clearing senescent macrophages in KRAS-driven lung cancer mice significantly improved tumorigenesis and progression, indicating that senescent macrophages contribute to lung cancer development [14, 15]. It is now well-established that the role of macrophages in tumors is largely determined by their polarization state. The best-characterized phenotypes are the classically activated (M1) and alternatively activated (M2) states [16]. M1 polarization is associated with pro-inflammatory responses against bacteria and viruses, as well as anti-tumorigenic effects, whereas M2 polarization is linked to anti-inflammatory and pro-tumorigenic responses. However, whether senescent TAMs also exhibit polarized phenotypes remains poorly understood.

As previously mentioned, studies on macrophage senescence have been conducted in both central nervous system and respiratory system tumors. However, whether macrophages in liver cancer undergo senescence remains to be unexplored, especially hepatocellular carcinoma (HCC) which accounting for 75%- 85% of all liver cancer cases [17]. Based on this, we established an in vitro co-culture model of murine hepatocellular carcinoma cell line Hepa1-6 and macrophages-like transformed murine cell line RAW264.7 to investigate and prove macrophages senescence under the influence of HCC cells. Furthermore, we will explore the impact of senescence on macrophage phenotypes, providing new evidence for understanding the immune landscape of hepatocellular carcinoma.

## Materials and methods

### Cell culture

Macrophage-like transformed murine cell line RAW 264.7, murine hepatoma cell line Hepa 1–6, Human Monocytic Leukemia Cell Line 1 (THP1), Human Hepatocarcinoma Cells line Hep 3B and Huh7 were obtained from the Chinese Academy of Sciences (Shanghai, China) and cultured at a density of 5 × 10^3^ cells per well in 12-well tissue culture-treated polystyrene plates. Cultivation was carried out in Dulbecco’s Modified Eagle Medium (DMEM) or Roswell Park Memorial Institute 1640 (RPMI-1640) supplemented with 10% fetal bovine serum (FBS), 100 U/mL of penicillin, and 100 mg/mL of streptomycin, at 37 ℃ in a humidified incubator containing 5% CO_2_. All cells were used below passage 10 after purchase. Media was completely refreshed every two days. Murine Bone Marrow-Derived Macrophages (BMDM) and murine T lymphocyte CTLL-2 were obtained from Procell system (Wuhan, China). Cultivation of CTLL-2 was carried out in RPMI 1640 + 1%Glutamax + 100 U/ml mouse Recombinant IL-2 + 10% FBS + 1%P/S, at 37 ℃ in a humidified incubator containing 5% CO_2_.

### Indirect co-culture of RAW 264.7/BMDM and Hepa 1–6

To assess the impact of cell-cell contact on macrophages senescence, indirect co-culture experiment is performed using Transwell chambers with a 0.4 μm pore size membrane. Culture 5 × 10^3^ Hepa 1–6 on the top and 5 × 10^3^ RAW 264.7/1 × 10^5^ BMDM in the bottom of transwell chambers in separate plates. Each chamber was cultured with complete medium at 37 ℃ in a humidified incubator containing 5% CO_2_ for durations of 3, 5, 7, and 10 days successively.

### Indirect co-culture of THP1 and Hep 3B, Huh7

THP-1 cells were seeded at a density of 5 × 10⁵ cells/well in the lower chamber of a 12-well Transwell plate. After 48 h of stimulation with 100 mmol/L PMA, the cells adhered and spread, activating into M0-type macrophages [18]. Hep 3B and Huh7 cells were seeded at a density of 1 × 10^4^ cells/well in the upper chamber of the 12-well Transwell plate. Each chamber was cultured with complete medium at 37℃ in a humidified incubator containing 5% CO_2_.

### SA-β-gal staining

The SA-β-gal staining kit was purchased (Beyotime, C0602) and staining was performed based on the manufacturer’s protocol. The cell culture plates were washed in 1×PBS, fixed with a combination of 2% formaldehyde and 0.2% glutaraldehyde at 37℃ for 5 min, and stained with a freshly prepared staining solution (X-gal, Sigma) at 37℃ overnight. Finally, the images were captured using a microscope equipped with a digital camera (Olympus, Japan).

### Cell cycle analysis

Flow cytometry was used to evaluate the cell cycle distribution [19]. RAW 264.7 Cells were delicately detached using a cell scraper and collected into a centrifugal tube and washed with PBS thrice, followed by fixation with ice-cold 95% ethanol overnight at -20℃ then centrifugated at approximately 300× g for 3–5 min and washed with ice-cold PBS twice. Cells were stained with PI staining buffer (50 mg/mL) (C1052, Beyotime Biotechnology, Shanghai, China) in the dark for 30 min at room temperature and analyzed using a flow cytometer (Beckman, Brea, CA, USA).

### Real-time quantitative PCR

Real-Time Quantitative PCR (q-PCR) was used to analyzed the relative mRNA expression as previous study [19]. Using a total RNA kit (R6834, Omega, Norcross, GA, USA) to extracted the total RNA from control and cu-culture RAW264.7 cells. Initially, cells were lysed with a buffer, after which the supernatant was transferred to a filtration column for centrifugation. Subsequently, ethanol was introduced into the column for RNA binding. Post-centrifugation, the RNA was adhered to the column, which was then washed thrice and air-dried. Finally, DEPC-treated water was used to elute the RNA, and its concentration was quantified. The isolated RNA underwent reverse transcription using a reverse transcription kit (M1631, Thermo Fisher Scientific, Waltham, MA, USA). The total RNA extracted was mixed with RT Buffer Mix, RT Enzyme Mix, and water, in adherence to the provided protocol. The reaction was incubated at 42 ℃ for 16 min to facilitate the formation of cDNA. For the amplification, forty PCR cycles were conducted using the SYBR Green Real-Time PCR Master Mix (11762100, Thermo Fisher Scientific, Waltham, MA, USA) in the Roche Light Cycler 480 system. The primers utilized for real-time PCR are listed in Table 1. Fluorescence-quantitative PCR was performed using a 20 µL reaction system, and the 2−∆∆Ct method was used to quantify the relative expression levels.

**Table 1:** Primer sequence

Genes	Forward primer	Reverse primer
P16	TCACACGACTGGGCGATTG	TGCCCATCATCATCACCTGAATC
P21	TCGCTGTCTTGCACTCTGGTGT	CCAATCTGCGCTTGGAGTGATAG
CD80	CCCCAGAAGACCCTCCTGATAGC	TGATGACAACGATGACGACGACTG
CD86	AGATCCAAGAGCCACTCCTACCTG	AGTGCTGCCTGCTAAGTTGTGAC
CD206	TGATTGGTGGCAATTCACGAGAGG	AACAGGCAGGGAAGGGTCAGTC
CD163	AATCACATCATGGCACAGGTCACC	TCGTCGCTTCAGAGTCCACAGG
ARG1	AAGACAGCAGAGGAGGTGAAGAG	GGTAGTCAGTCCCTGGCTTATGG
IL1α	GATTCTGAAGAAGAGACGGCTGAG	TCTGGTAGGTGTAAGGTGCTGATC
IL1β	AATCTCGCAGCAGCACATCAAC	AGGTCCACGGGAAAGACACAG
IL6	CGGAGAGGAGACTTCACAGAGG	TTCCACGATTTCCCAGAGAACATG
IL10	GCTCCAAGACCAAGGTGTCT	AGGACACCATAGCAAAGGGC
ISG15	CAAGCAGCCAGAAGCAGACTCC	CGGACACCAGGAAATCGTTACCC
TGFβ1	ACTGGAGTTGTACGGCAGTG	GTATTCCGTCTCCTTGGTTCAGC
MMP3	AGAAGGAGGCAGCAGAGAACC	GTAGGATGAGCACACAACCACAC
IGFBP3	TGAGTCCGAGGAGGAGCACAATG	GGCATGGAGTGGATGGAACTTGG

### Immunofluorescence microscopy

Polystyrene plates were wash with PBS, fixed with 4% paraformaldehyde for 10 min, then incubated with anti-p16, p21, PCNA, LMNB1 primary antibodies overnight at 4℃. On next day, trice 2 min PBS washes followed by 2-hour incubation at 37℃ with fluorescence-labeled secondary antibodies. After thrice 2 min PBS washes, nuclei were counterstained with DAPI. Images were captured using a ZEISS LSM 980 confocal microscope.

### Western blotting

Western blotting was used to detect protein expression as previous study [19]. Cell-derived protein samples were prepared using RIPA buffer (P0013B, Beyotime, Shanghai, China) supplemented with a cocktail inhibitor (11697498001, Roche, Munich, Germany) and subjected to a 15 min boiling process. 10 µg of each sample was loaded onto an SDS-polyacrylamide gel. After SDS-PAGE, proteins were transferred from the gel to a polyvinylidene fluoride (PVDF) membrane. Following a 2 h blocking step with 10% fat-free milk or bovine serum albumin (for phosphorylated proteins) at room temperature, the membrane was incubated overnight at 4 ℃ with primary antibodies. The antibodies used were p21(ab188224, Abcam, Cambridge, UK), γ-Histone H2A.x(ab81299, Abcam, Cambridge, UK). After washing with TBS-T, secondary antibodies (RS0002, ImmunoWay, Shanghai, China) were applied for 1 h. The target proteins were then visualized using an ECL Western Blotting substrate and detected with an Odyssey Imaging System (Li-COR, Lincoln, NE, USA).

### ELISA for the analysis of secreted cytokines

The concentrations of cytokines (IL1β, IL6, IL10, TGF-β and MMP3) were determined using ELISA kits, following the manufacturer’s instructions. After 24 h of co-culture, cell culture media from different treatment groups were collected, appropriately diluted, and analyzed using a microplate reader [19].

### Bulk RNA-seq

Using a total RNA kit (R6834, Omega, Norcross, GA, USA) to extracted the total RNA from control and cu-culture RAW264.7 cells. RNA purification, reverse transcription, library construction and sequencing were performed at Shanghai Majorbio Bio-pharm Biotechnology Co., Ltd. (Shanghai, China) according to the manufacturer’s instructions (Illumina, San Diego, CA). The transcriptome library for RNA-seq was generated by using 1 µg of total RNA with the Illumina^®^ Stranded mRNA Prep, Ligation kit from Illumina (San Diego, CA). After quality control of raw reads, the clean reads were mapped using HISAT2 [20] software and further assembled by StringTiein. Differentially expressed genes were performed using the application of DESeq2 [21] (version 1.41.8) employing a threshold based on a Benjamini-Hochberg adjusted *p*-value of 0.05 and an absolute fold change of > 2 (|Log2FC| >1).

### Phagocytosis assay

Phagocytosis was measured in both senescent and control cells. Red fluorescent polystyrene beads (diameter 1 mm, Tomicro Biotech) at a dilution of 0.5 ml stock to 100 ml media were added to macrophage cultures and incubated at 37℃ with 5% supplemental CO_2_ for 12 h. After this incubation time, cells were washed to remove any beads not internalized and fixed with 4% paraformaldehyde [22] (PFA, Biosharp, Beijing, China). Nuclei were counterstained with DAPI. Using Image J the area of the cell occupied by beads was divided by the total cell area to determine the percentage of beads occupying the cells in each frame.

### Flow cytometry

For flow cytometry staining, dissociated cells were incubated with Cell Staining Buffer (BioLegend, BD) followed by staining with primary antibodies: anti-mouse CD45 (BD, 103, 130),F4/80 (eBioscience, 11-4801-82), MHC Class II (I-A/I-E) (eBioscience, 11-0862-82), CD86(eBioscience, 12-0862-82), CD206(eBioscience, MA5-16870). Then PBS wash for remove the anti-body extracellular and analyzed using a flow cytometer (Beckman, Brea, CA, USA).

### Co-culture with T cell

To investigate the effect of senescent macrophages on T cell function, we used a Transwell chamber for co-culture. RAW264.7 cells co-cultured with Hepa 1–6 cells served as the experimental group, while RAW264.7 cells without co-culture served as the control group. CTLL-2 cells were then added to both groups of macrophages for direct co-culture. The suspended T lymphocytes were collected, centrifuged to remove the old culture medium, washed twice with 1× PBS by centrifugation, and resuspended. The cell concentration was adjusted to 3 × 10⁵ cells/ml. 100 µl of Membrane Permeabilization Solution A and incubate for 6 min to fix the specimen cells, then wash once with PBS, and centrifuge at 1300 rpm for 5 min. Sequentially, 50 µl of Membrane Permeabilization Solution B and the intracellular antibodies, GRB-FITC and Perforin-APC incubate for 15 min in the dark. Terminate the reaction, and centrifuge at 1300 rpm for 5 min. Wash the cells twice with PBS. After washing, discard the supernatant, resuspend the cells in 1 ml of PBS and proceed with instrument analysis.

### Sudan III staining

To exam the lipid metabolism of macrophages, we conducted Sudan III Staining. Culture plates were washed twice by PBS. 10 min 4% paraformaldehyde fixation follow by 15 min Sudan III Staining. The images were captured using a microscope equipped with a digital camera (Olympus, Japan).

### Transwell invasion assay

Transwell chambers were used for invasion assay [19]. For control group, the culture medium without any cells supplemented with 10% FBS was in the lower chamber, then a single-cell suspension of 4 × 10^4^ Hepa 1–6 cells were seeded into the upper chamber on Matrigel for invasion evaluation. For experimental group, RAW264.7 cells were seed in the lower chamber with complete medium, then a single-cell suspension of 4 × 10^4^ Hepa 1-6cells was seeded into the upper chamber on Matrigel. Following 24 h of incubation, the cells in the upper chamber were fixed in 4% paraformaldehyde for 5 min and stained with 0.2% crystal violet for another 5 min, followed by two PBS washes. The migrated cells on the membrane were then quantified using microscopic examination.

### Statistical analysis

The results were presented as the Mean ± SD from three independent biological replicates. Differences between two groups were conducted using the Student’s t-test or Welch’s t-test. Statistical significance was determined with a *p*-value of less than 0.05. The statistical software used Prism 10.

## Results

### Co-culture group exhibited prominent morphological alteration and stained positively for senescence-associated ß-galactosidase (SA-ß-Gal)

**Fig. 1:**
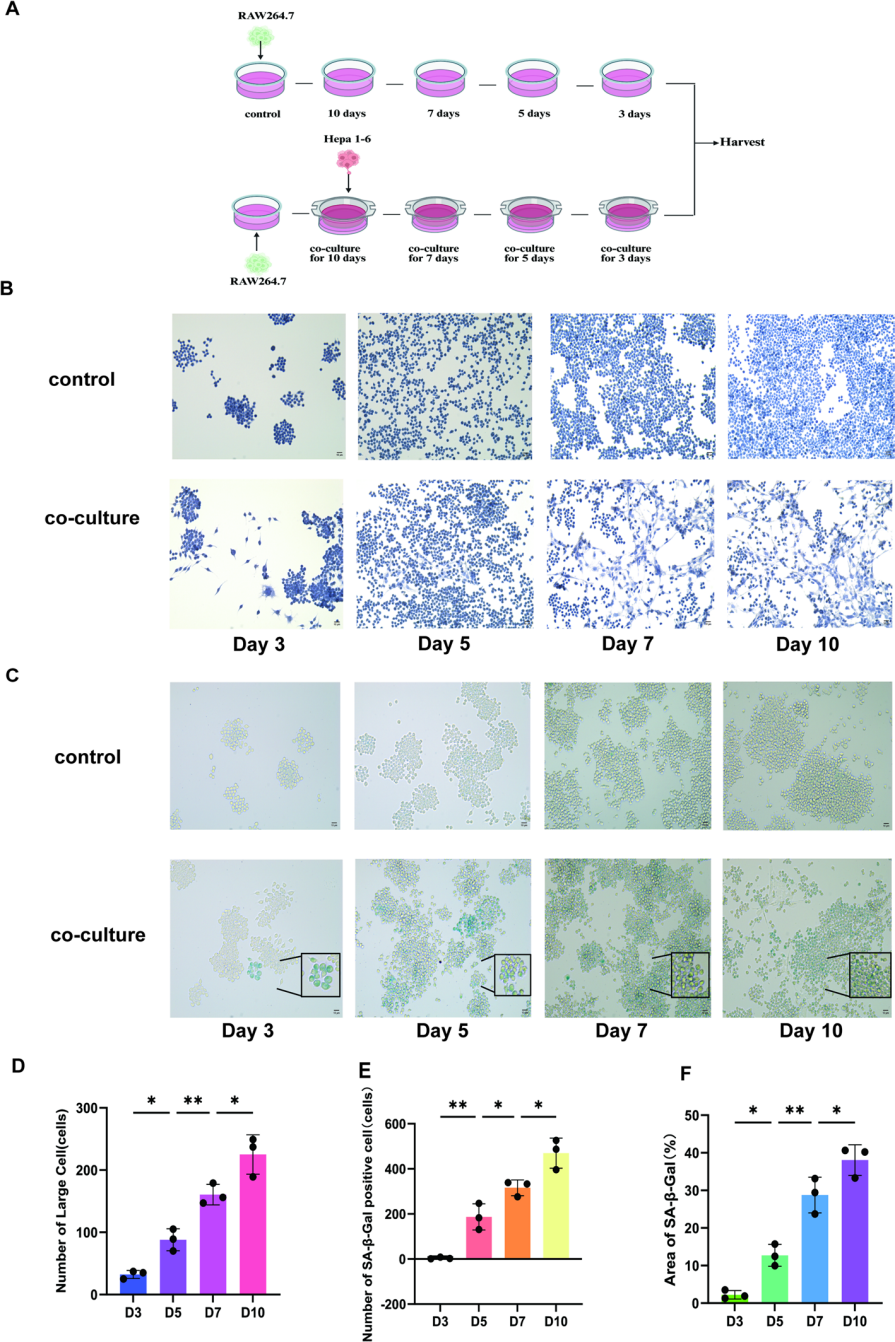
Comparison of morphology and Sa-β-Gal staining in control group and co-culture group on day 3,5,7,10. **A** Schematic illustration of the indirect co-culture system. **B** The representative images of morphology in two group on day 3,5,7,10. **C** Sa-β-Gal staining, respectively in two group on day 3,5,7,10. close-up, high-power magnification. **D** The quantification of Large cell number in co-culture group on day 3,5,7,10. **E** The quantification of Sa-β-Gal positve cell number in co-culture group on day 3,5,7,10. **F** The quantification of Sa-β-Gal staining positive area in co-culture group on day 3,5,7,10. Significance is noted as * *p* < 0.05, ** *p* < 0.01, and *** *p* < 0.001, **** *P* < 0.0001, *ns* no significance

When the state of macrophages changes or polarization occurs, their morphology also undergoes corresponding alterations. To observe whether morphology of RAW264.7 change after co-culture with Hepa 1–6 cells (Fig. 1A), we employed hematoxylin staining to better show the shape of cells, then observed the morphology of RAW264.7 after 3, 5, 7, and 10 days. Compared with the control group, we found that on day 3 of co-culture, the body of RAW264.7 cells and their nuclei began to increase in size and cytoplasm elongated. Then as time passed, the number of such cells gradually increased (Fig. 1B). Consistent with the morphological changes, the staining of the senescence-associated marker SA-β-Gal also increased (Fig. 1C). Different cultured densities of RAW264.7 got a same result (Fig. S1).

### Elevated proportion of co-culture RAW264.7 in G1 phase

**Fig. 2:**
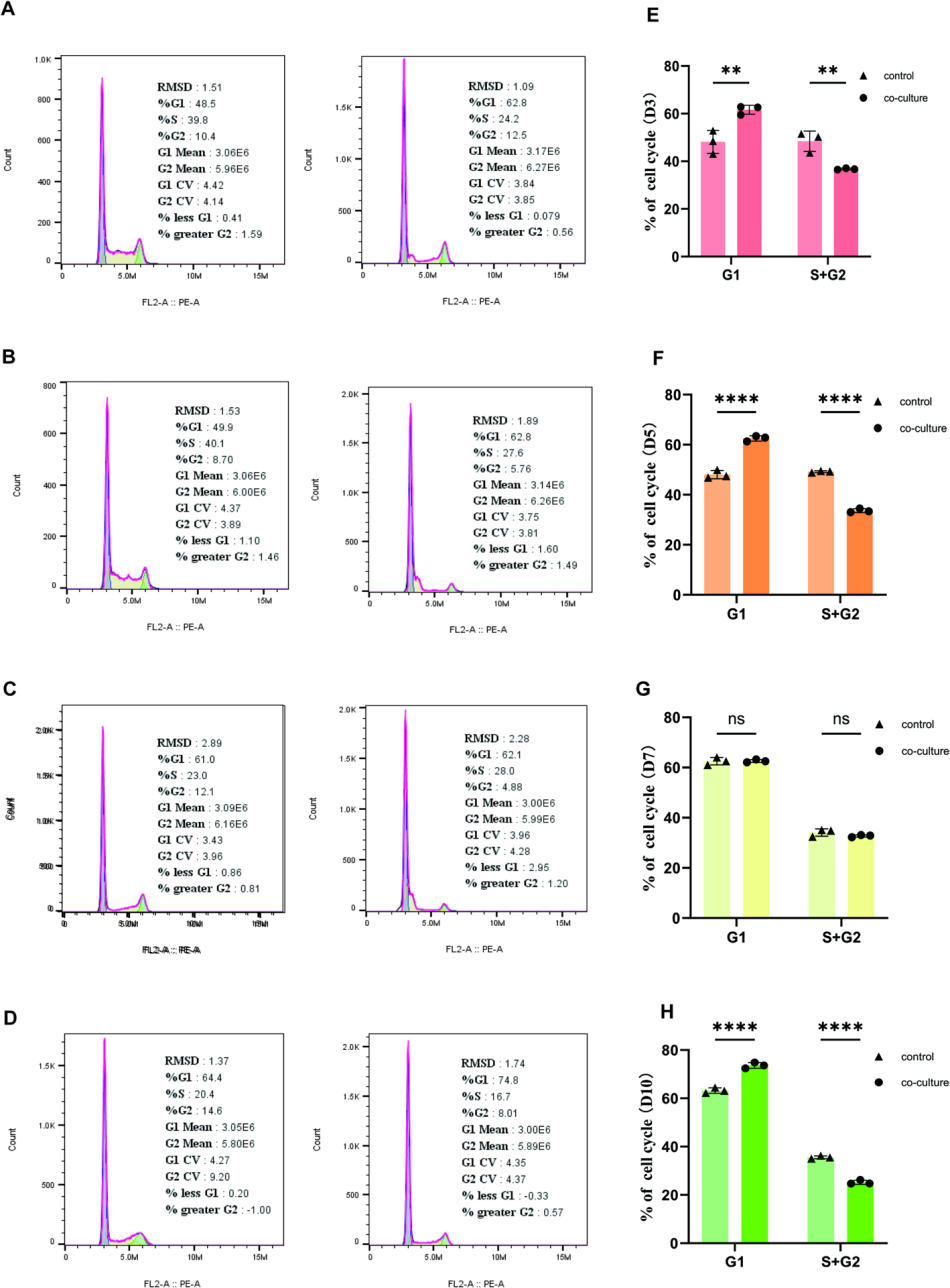
Assessment of cell cycle distribution using PI staining day 3,5,7,10 in two group. **A**–**D** Distribution of cell cycle in two group on day 3,5,7,10, respectively. **E**–**H** The quantification of G1 and S + G2 stage distribution in two group on day 3,5,7,10, respectively. The values represent the mean ± SD of three independent experiments. * *p* < 0.05, ** *p* < 0.01, *** *p* < 0.001, *ns* no significance. *CTRL* control, *CO-C* co-cultur

Morphological alteration alone cannot define the occurrence of senescence. Therefore, we hope to confirm senescence through its basic characteristic- the cell cycle arrest. So, we continued to perform cell cycle analysis using flow cytometry on the control and co-culture groups on day 3, 5, 7, and 10 after co-culture (Fig. 2A-D). We observed that, starting from day 3, the proportion of cells in the G1 phase was significantly higher in the co-culture group compared to the control group (Fig. 2E, F), while the proportion of cells in the S and G2 phases gradually decreased. Although no statistically significant difference in the G1 phase was observed between these two groups on day 7 (Fig. 2G), but on day 10 the proportion of G1 phase in the co-culture group increased significantly again (Fig. 2H).

### Bulk RNA sequencing analysis showed co-cultured RAW264.7 cell lines enhanced the expression of senescence-related genes

**Fig. 3:**
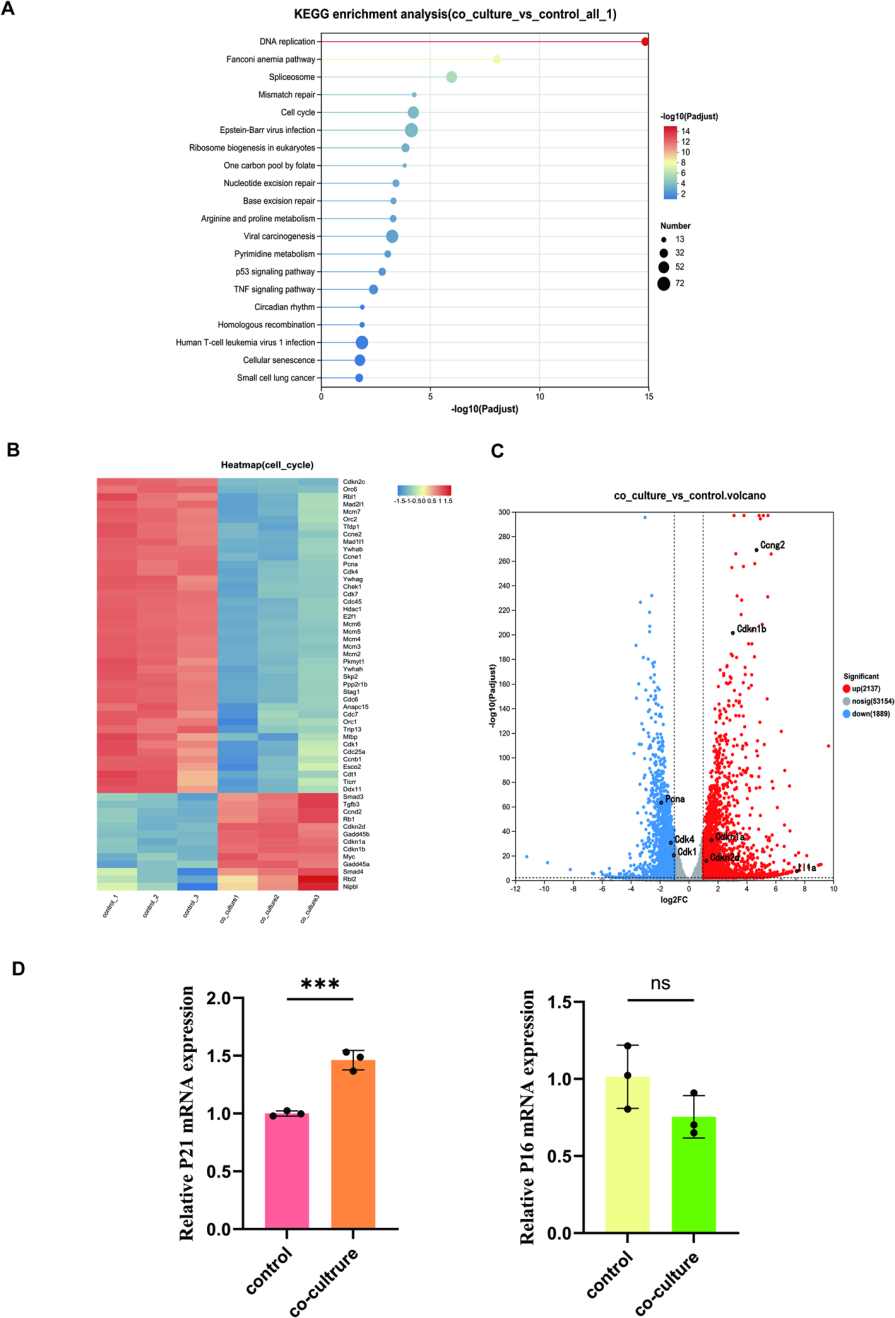
Bulk RNA-seq analysis and detection of cyclin-dependent kinase inhibitor and cell cycle relative proteins. **A** Pathway analysis based on RNA-seq data, highlighting upregulated pathways in control group and co-culture group. **B **Heatmap of scaled gene expression changes in genes associated with cell cycle pathway in two groups. **C** Volcano plots illustrating protein expression changes between two groups. **D** P21 and P16 mRNA expression detected by q-PCR. The values represent the mean ± SD of three independent experiments. * *p* < 0.05, ** *p* < 0.01, and *** *p* < 0.001, **** *P* < 0.0001, *ns* no significance

To better understand the major molecular pathological features, we compared co-cultured RAW264.7 and control group using Bulk RNA-seq. In order to rule out the possible confounding effects of density-induced senescence in RAW cells, we conducted Bulk RNA-seq and subsequent experiments on the cells that co-cultured for 5 days. We found that co-cultured RAW264.7 were presented with relationship with DNA repair, cell cycle and cell senescence pathways (Fig. 3A). Then the differential genes analysis indicated that co-culture RAW264.7 contained significantly enhancing expression of senescence-related genes, such as CDKN2A, CDKN1B, CDKN2D, CCNG2, while repressing the expression of proliferation-related or cell cycle -related genes, such as PCNA, CDK1, CDK4 (Fig. 3B, C). qPCR results also confirmed that in co-culture group, p21 protein which encoded by CDKN2A gene was up-regulated, but there was no statistical significance in p16 expression between two group (Fig. 3D).

### The co-culture group exhibited senescence characteristics

**Fig. 4:**
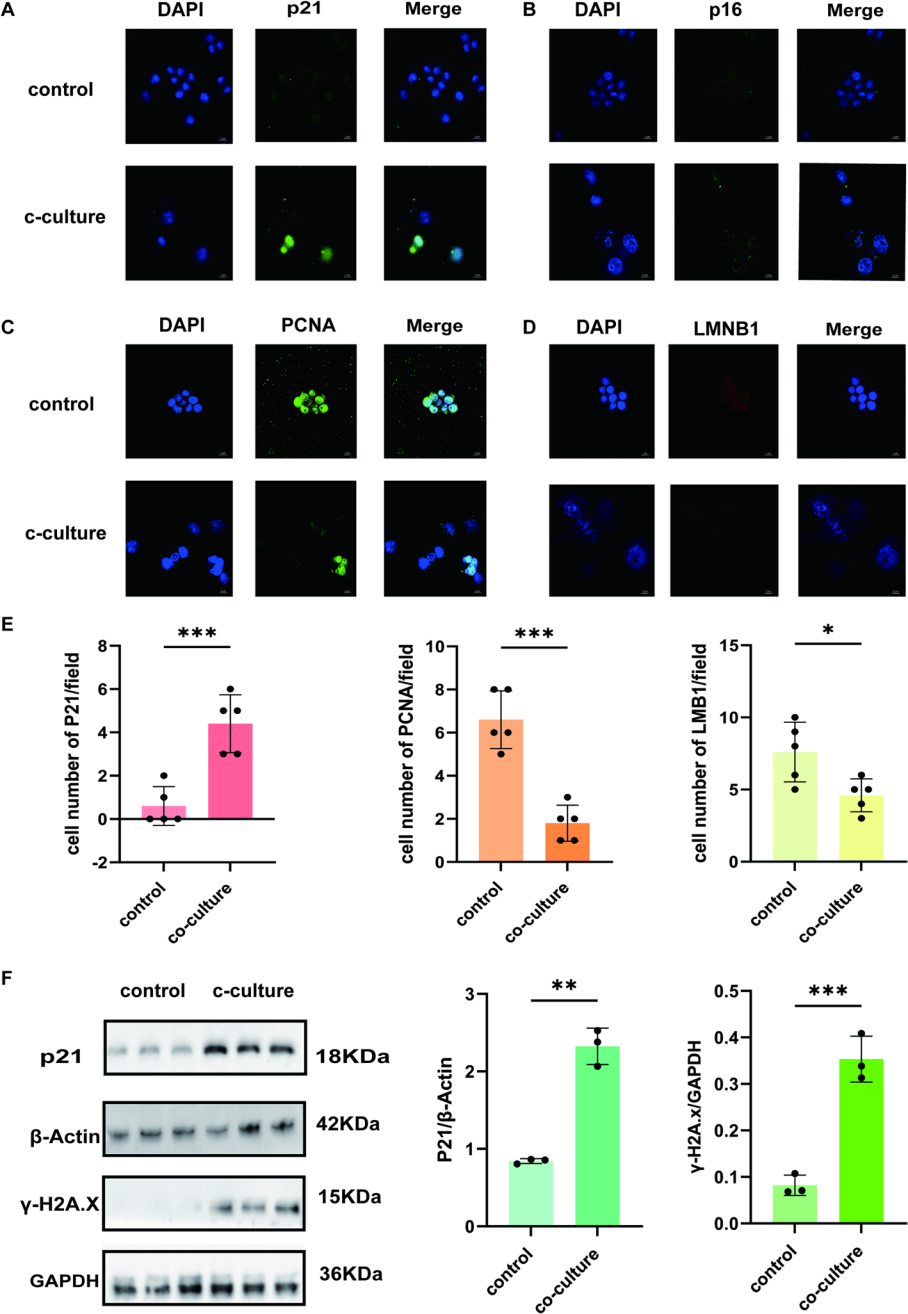
**A** p21 expression in control group and co-culture group was detected by immunofluorescence, Scale bars 2 μm. **B** P16 expression in control group and co-culture group was detected by immunofluorescence, Scale bars 2 μm. **C** PCNA expression in control group and co-culture group was detected by immunofluorescence, Scale bars 2 μm. **D** LMNB1 expression in control group and co-culture group was detected by immunofluorescence, Scale bars 2 μm. **E** The quantification of expression of P21, PCNA and LMNB1 in two group. **F** Western blotting was utilized to evaluate protein expression levels of γ-H2A.x and P21. The values represent the mean ± SD of three independent experiments. * *p* < 0.05, ** *p* < 0.01, and *** *p* < 0.001, **** *P* < 0.0001, *ns* no significance

Immunofluorescence staining in two group, which revealed enhanced p21 expression in co-culture group (Fig. 4B) but not p16 (Fig. 4A). We also performed immunofluorescence staining with PCNA, the protein involved cell cycle progression, and LMNB1 an suitable marker of nuclear envelope which ensures structural integrity and chromatin stability and reduces while cellular senescence. We found that PCNA (Fig. 4C) and LMNB1 (Fig. 4E) staining were weak and negative in co-culture group compared with control group by confocal microscopy. Western blotting also show the high protein expression of p21 and γ-H2A.x which the most robust DNA damage response(DDR)marker and senescence marker (Fig. 4F). In co-culture model of BMDM with Hepa 1–6 and human-derived macrophage cell line THP1 with Hep 3B and Huh7 cells, we also observed that the expression of p21 protein levels in co-culture groups was higher than that control group (Fig. S2A-D).

**Fig. 5:**
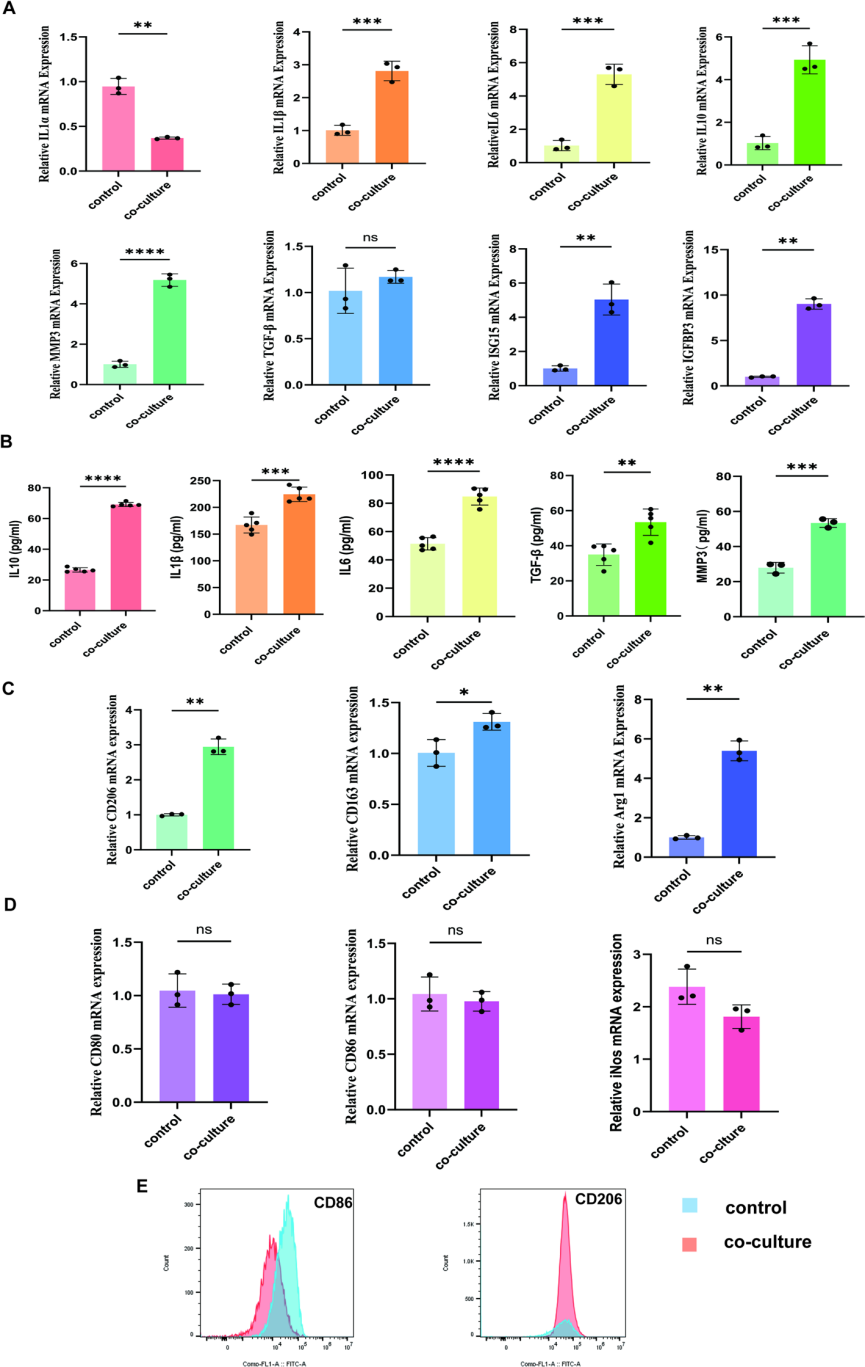
Comparison of SASP relatived genes expression and polarization in two group. **A** Detection of SASP relatived genes, including IL1α, IL1β, IL6, IL10, MMP3, TGFβ, ISG15, IGFBP3. **B** Detection of cytokines IL10, IL1β, IL6 and TGF-β by ELISA. **C** The relative mRNA expression levels of CD206, CD163 and Arg1 were measured to assess the impact of co-culture on macrophages M2 polarization compared to the control group. **D** The relatived mRNA expression levels of CD80, CD86 and iNos were measured to assess the impact of co-culture on macrophages M1 polarization compared to the control group. The values represent the mean ± SD of three independent experiments. * *p* < 0.05, ** *p* < 0.01, and *** *p* < 0.001, **** *P* < 0.0001, *ns* no significance

We performed q-PCR for well-established biomarkers of senescence, including pro-inflammatory factors that are linked senescence-associated secretory phenotype (SASP) on RNA extracted from co-culture RAW264.7 and control group. We observed an increased expression of pro-inflammatory SASP: IL6, IL1α, IL1β, MMP3 and P53-21 axis associated SASP ISG15 and IGFBP3 were also elevated in co-culture group. But another classical SASP TGFβ was no significantly difference reduced in expression (Fig. 5A). Then we employed ELISA to evaluate the levels of cytokines IL10, IL1β, IL6, TGF-β and MMP3, the results show that expression levels of the aforementioned conventional SASP proteins were higher in the co-culture group compared to the control group. (Fig. 5B).

### Co-culture promotes the polarization of M0 macrophages to an M2 phenotype

The expression of CD206 and CD163, which are recognized as M2 macrophages markers, while CD80, CD86 and iNos are M1 macrophages marker. In the results of qPCR, the expression of CD206 and CD163 were significantly upregulated in co-culture group. The expression of M2-relate genes such as Arg1 was up-regulate too (Fig. 5C). The expression of CD80, CD86 and iNos showed a non-significant upregulation (Fig. 5D). We also detected the expression of protein CD86 and CD206 by flow cytometry, and the results were consistent with those obtained by q-PCR (Fig. 5E).

### The functions, metabolism of co-culture macrophages had changed

**Fig. 6:**
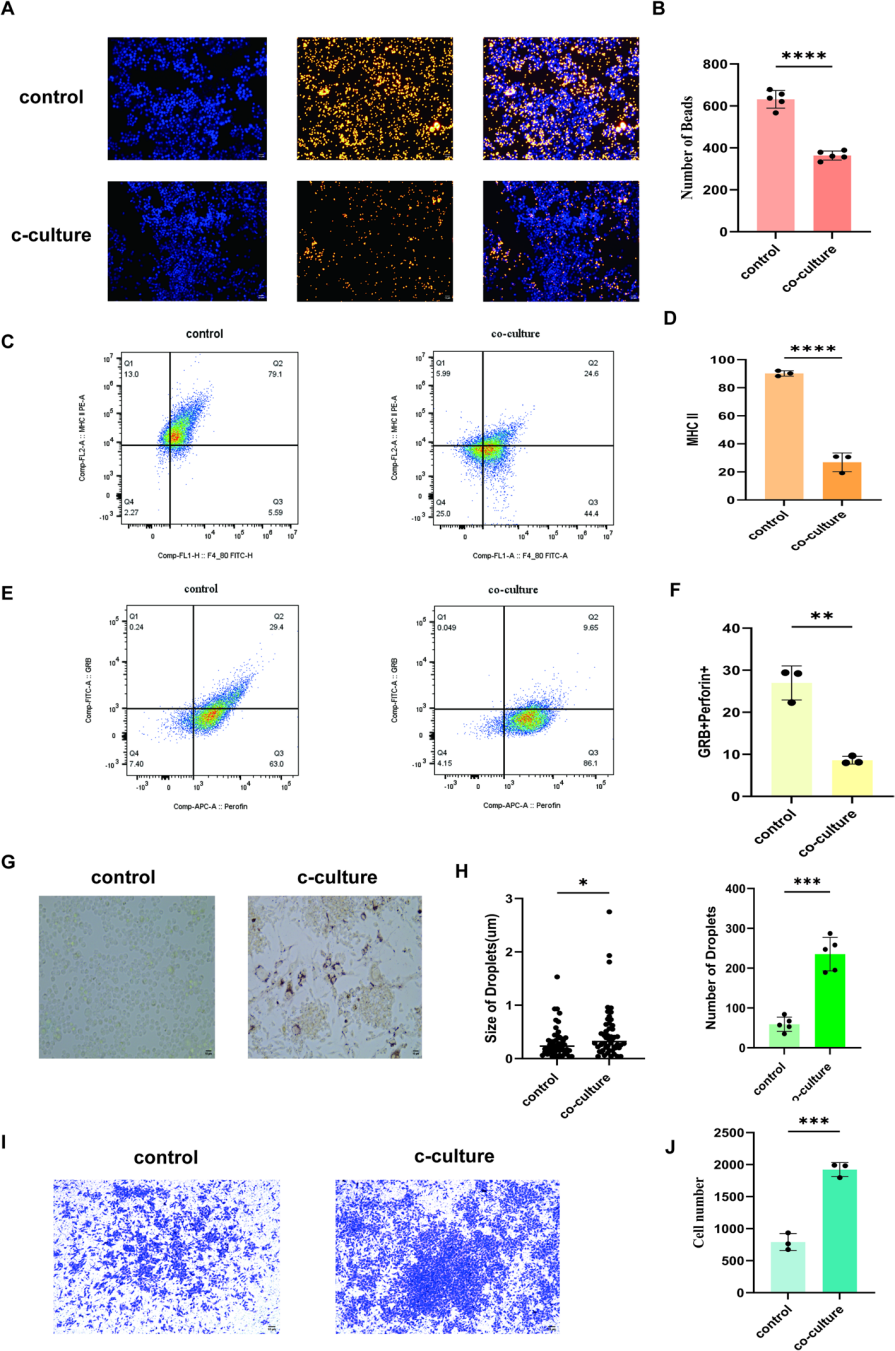
Functional comparison between control group and co-culture group. **A** Image of macrophages with internalized beads in control group and co-culture group, **B** and the results of the quantitative analysis are shown on the right. **C** The expression of MHC Class II was assessed by flow cytometry, **D** and the results of the quantitative analysis are shown on the right. **E** the expression of granzyme B and perforin in T lymphocytes. **F** and the results of the quantitative analysis are shown on the right. **G** Sudan III Staining showed the lipid droplets in the cytoplasm of macrophages in two groups. **H** Quantitative analysis of droplets in size and number. **I** The effect of RAW264,7 and Hepa 1–6 co-culture on the invasion of Hepa 1–6 was assessed by invasion assay, **J** and the results of the quantitative analysis are shown on the right .The values represent the mean ± SD of three independent experiments.* *p* < 0.05, ** *p* < 0.01, and *** *p* < 0.001, **** *P* < 0.0001, *ns* no signiffcance

The functions of senescent macrophages will change [22]. We conducted phagocytose, flow cytometry, Sudan III Staining and invasive assay to assess the ability of macrophages. The results showed co-culture RAW 264.7 and BMDM phagocytized less beads under the same culture conditions with control group, revealed the ability reduction of phagocytose (Fig. 6A, B, Fig. S2E, F). Then we employed flow cytometry to demonstrate co-cultured RAW264.7 and BMDM downregulating MHC Class II expression (Fig. 6C, D, Fig.S2 G, H). As a critical link between innate immunity and adaptive immunity, we propose that macrophage senescence influences T cell function. Therefore, we employed direct co-culture of macrophages and T cells to assess the changes in T cell function, and the result showed that the levels of the cytotoxic molecules perforin and granzyme B in T lymphocytes were significantly reduced in the group co-cultured with tumor cells, indicating that T cell function was attenuated compared with the control group (Fig. 6E, F). Lipid metabolic abnormality was one of symbol in senescence metabolism. We performed Sudan III Staining to detected the lipid droplets in RAW264.7 and there were more variably sized lipid droplets in the cytoplasm of co-cultured macrophages (Fig. 6G, H). Furthermore, we observed a direct correlation between co-culture and a consequential increase in the invasive ability of Hepa 1–6 cells (Fig. 6I, J).

## Discussion

Hepatocellular carcinoma (HCC) is the most common subtype of liver cancer. Since early-stage HCC often presents with nonspecific symptoms, most patients miss the opportunity for timely intervention. Despite the emergence of various treatments, the outcomes for advanced-stage patients remain unsatisfactory. Compared to targeted therapy, immunotherapy demonstrates superior efficacy and fewer side effects in treating advanced cases. Furthermore, depth exploration of HCC immune microenvironment can discover more pathways and approaches for immunotherapy.

Macrophages are one of the key components of the liver and immune system. When HCC occurs, changes inevitably happen in the macrophages. We hypothesize that during their growth, HCC cells secrete various inflammatory factors, including the aforementioned classic senescence-associated secretory phenotype (SASP) components [23–25], which induce macrophages senescence. However, macrophages exhibit unique structural and functional characteristics compared to other cell types, for instance, lysosomes in macrophages cytoplasm may elevate the staining of β-galactosidase. Additionally, macrophages can also secrete pro-inflammatory factors in a non-senescent state, many of them were SASP too. These characteristics overlap with features of cellular senescence that increases the challenge of detecting macrophage senescence; Therefore, we believe that the term “senescence-like” is more suitable for describing the state of co-cultured macrophages in our research. Due to the heterogeneity of cellular senescence, there remains no specific assay to identify senescent cells, relying on senescence associate β-galactosidase staining (SA-β-Gal) staining or SASP assays alone may not fully reflect the real senescent state of macrophages. Base on this, we established an indirect co-culture model of HCC cells with macrophages and adopted multiple methods, including SASP detection and SA-β-gal to investigate whether macrophages in HCC microenvironment undergo senescence-like change.

From our co-culture model, we observed that starting on day 3, cell bodies became fatted, nuclei were enlarged, the number of these macrophages progressively increased over time. SA-β-gal staining outcomes aligned with the observed alterations in morphology. However, Hall proposed that SA-β-gal activity can be induced in macrophages and is not exclusive to senescence [26]. To further validate our hypothesis, we performed cell cycle analysi**s** and found that only the day 7 co-culture group showed no significant difference in the proportion of G1-phase cells compared to the control. We speculate that as RAW264.7 cells reached higher densities during culture, a subset exhibit senescence, which aligns with Holt’s findings [22]. Nevertheless, over extended time periods, the proportion of G1-phase cells in the co-culture group increased markedly again, suggesting that tumor cells may play a critical role in this process. The microenvironment formed by tumor cells and the cytokines they secrete may accelerate cell cycle arrest. In order to rule out the possible confounding effects of density-induced senescence in RAW cells, we conducted follow-up experiments on day 5 co-culture cells.

Mechanisms of cellular senescence so call cell cycle arrest are mediated through alteration of cell cycle regulatory pathways, particularly upregulation of the cyclin-dependent kinase inhibitor proteins, including p16 and p21, which are currently recognized as hallmarks of cell cycle arrest. These regulators can act individually or sequentially to control cell cycle progression [27–30]. In bulk RNA-seq analysis we can see the up-regulation mRNA of CDKN1A which encode protein p21 and KEGG enrichment analysis revealed significant enrichment of the DNA replication pathway, suggesting the presence of exogenous or endogenous stress that may cause DNA damage and subsequently activate repair programs. γ-H2A.X is the most robust DNA damage response marker [31] which up expressed in co-culture group. Key pathways including the Fanconi anemia pathway and mismatch repair [32, 33]—both well-established components of the DNA damage response—were notably involved. The DNA damage response generally leads to two cellular outcomes [34]: cell death if the damage is irreparable, or cell cycle arrest if the damage is moderate and repairable. The results showed significant enrichment in cell cycle-related pathways, as well as discernible changes in cellular senescence pathways. It was demonstrated that under the condition of co-culture with tumor cells, macrophages underwent senescence-like change. These findings were further validated through qPCR and immunofluorescence staining. Meanwhile, in our experimental group, the expression of lamin B1 and proliferating cell nuclear antigen (PCNA) were decreased or absent, which can observe during cellular senescence [35], suggests that co-culture macrophages exhibit feature of senescence in cell cycle regulation. Nonetheless, our research did not demonstrate any notable variance in the levels of p16 between two cohorts, this might be related to the way of senescence. The molecular mechanisms underlying cellular senescence can be categorized into two main classes: DNA damage-induced senescence and non-DNA damage-induced senescence. p16 was the regulating key molecule in non-DNA damage mechanisms [36, 37]. In this study, RNA-seq data and Western blotting result showed that there was DNA damage existed in co-culture group. On the other side, the indirect co-culture system mimics the SASP microenvironment which can activate DNA damage response(DDR) through localized inflammation and is recognized as one of the key factors inducing cellular senescence [38]. SASP also can operate multiple signaling pathway upstream, for instance, TGF-β activate SMADs pathway and PI3K-AKT pathway, IL6 activate JAK-STAT3 pathway, all these pathway may enhance the expression of p21 [39, 40]. We considered that HCC induced macrophage senescence through the up expression of p21 primarily via SASP. Additionally, p21 plays a role in the regulation of macrophage polarization. Tumor cells continuously release pro-inflammatory factors, creating an inflammatory environment around the tumor [41, 42]. Senescent macrophages can promote M1 to M2 phenotype reprogramming via p21 by downmodulating IFN-β production [43], thereby suppressing inflammation but reducing the tumor-suppressive function of macrophages simultaneously. Consistent with this, in co-culture group, macrophages displayed an M2-like phenotype. This indicates that through gambling with immune cells, hepatocellular carcinoma can induce immune cell senescence-like change to acquired M2-like macrophage function, thereby sustaining its own proliferation or immune escape. Here, we have to mention the results of SASP detection. Classic SASP associated gene such as IL1β, IL6 which upregulated in our study were also the inflammatory factor secreted by macrophages in non-senescent contexts. Therefore, such factors may lack specificity in validating macrophage senescence. Based on this, we selected the p21-associated SASP factors ISG15 and IGFBP3 for further investigation and the results supported our hypothesis.

The alterations of senescence-like macrophage involve not only phenotype but also function. Phagocytosis is the most direct and crucial function of macrophages as the first line of innate immunity. Some studies have shown that senescence compromises the ability of macrophage, such as antigen presenting, phagocytosis, and cytokine secretion [44, 45]. Scholars believe that immune cell senescence is a manifestation of organismal or immune system aging [46, 47], In our study, HCC acts as a catalyst of immune system aging which promotes the senescence of immune cells. Furthermore, we observed a reduction expression of MHC class II of co-cultured macrophages. Previous studies have showed that tumors can reduce MHC class II expression in macrophages through prostaglandin E2 (PGE2) secretion, impairing their antigen-presenting capacity while promoting the expansion of Arg1 + macrophages [48, 49], this is also consistent with our findings that Arginase 1 (Arg1) expression was increased. Arg1 not only an indicator of M2 polarization in tumor-associated macrophages (TAMs) [50]. Zhu’s research revealed that, macrophages play a critical role in arginine-mediated breast cancer progression, meanwhile, these macrophages predominantly have a high level of arginine metabolic activity and exhibit an M2-like phenotype [51]. M2 macrophages utilize Arg1 to hydrolyze large amounts of imported arginine. Unlike the direct tumor-promoting effects of high arginine level, the primary function of arginine consumption by M2 macrophages is to restrict the availability of neighboring arginine-dependent cells, that’s why M2 macrophages can suppress T effectors by blocking their supply of arginine [52]. As the T cell suppression effect mentioned before, our research results also showed that the expression of granzyme B and perforin in T cells which co-culture with RAW254.7 and Hepa 1–6 was reduced, suggesting that T cell function might be inhibited. However, except the effect of arginine consumption, more interaction between senescent macrophages and T cells still requires further exploration. In addition to arginine metabolism, abnormality of lipid metabolism has also been implicated in multicellular senescence and can further induce senescence and functional impairment in cells [53–56]. Supporting this, we observed an accumulation of variably sized lipid droplets in the cytoplasm of co-cultured macrophages, providing indirect evidence that hepatocellular carcinoma associated macrophages undergo senescence-like change.

In addition to altering their own functions, senescence-change macrophages also can influence the tumor microenvironment through SASP, making tumor cells more aggressive. Matrix metalloproteinases(MMPs) are a well-established element of SASP component [6], known for.

degrade the basement membrane and extracellular matrix, which facilitate tumor invasion and metastasis. Our qPCR and ELISA results revealed upregulation of MMP3 related genes and protein in the co-culture group, and invasion assays show that Hepa1-6 cells co-cultured with macrophages demonstrated significantly enhanced invasive capability compared to tumor cells cultured alone.

The current limitation of this study is that it only elucidated the senescence-like phenomenon of macrophage induced by hepatocellular carcinoma, without further explaining the underlying mechanism. The focus of subsequent research is to further explore the mechanism by which hepatocellular carcinoma induces senescence-like change of macrophage and to validate it through in vivo experiments.

## Conclusion

In our in vitro indirect co-culture model, we employed various methods—including morphological observation, SA-β-Gal staining, cyclin-dependent kinase inhibitor p21 gene and protein detection, analysis of proliferation-related protein PCNA and senescence-associated protein Lamin B1, and SASP-related gene expression—to demonstrate that hepatocellular carcinoma cells can induce macrophage acquired senescence-like phenotype. Furthermore, tumor-induced senescence-like macrophages acquired M2-like phenotype. Additionally, senescence-like macrophages exhibited a tendency of promoting tumor invasion. These experimental results can serve as a foundation for our subsequent research on the senescence-related immune microenvironment in hepatocellular carcinoma, and for further exploring more information of the tumor environment in vivo.

## Supplementary Information

Below is the link to the electronic supplementary material.

Supplementary Material 1.

Supplementary Material 2.

## Data Availability

The datasets generated and/or analysed during the current study are available in the NCBI repository，BioProject: PRJNA1364196: macrophages senescence in vitro.

## Abbreviations

HCC: Hepatocellular carcinoma
SASP: Senescence-associated secretory phenotype
SA-β- Gal: Senescence-associated β-galactosidase
TNF-α: Tumor necrosis factor-α
IL-1α: Interleukin-1α
IL-1β: Interleukin-1β
IL-6: Interleukin-6
INF-γ: Interferon-γ
VEGF: Vascular endothelial growth factor
PD-L1: Programmed cell death ligand 1
Tim3 T: Cell immunoglobulin domain and mucin domain 3
TAM: Tumor-associated macrophage
TME: Tumor microenvironment
IL-10: Interleukin-10
TGF-β: Transforming growth factor-β
MMP3: Matrix metalloproteinase 3
ISG15: Interferon stimulated gene 15
IGFBP3: Insulin-like growth factor binding protein-3
Arg1: Arginase-1
GO: Gene ontology
KEGG: Kyoto encyclopedia of genes and genomes
DDR: DNA damage response
CDKN2A: Cyclin-dependent kinase inhibitor 2 A
CDKN1B: Cyclin-dependent kinase inhibitor 1B
CDKN2D: Cyclin-dependent kinase inhibitor 2D
PCNA: Proliferating cell nuclear antigen
CDK1: Cyclin dependent kinase 1
CDK4: Cyclin dependent kinase 4
LMNB1: Lamin B1
γ-H2A.X: Phosphorylated forms of H2A histone family member X
