# Supplementary material for: Induction of senescence like macrophages by hepatocellular carcinoma cells in vitro
Source: Discov Oncol. 2026 Jun 1;17:1117. doi: 10.1007/s12672-026-05348-8 (PMC13437835; doi:10.1007/s12672-026-05348-8)
Supplement: Supplementary file 1 [file 12672_2026_5348_MOESM2_ESM.pdf]

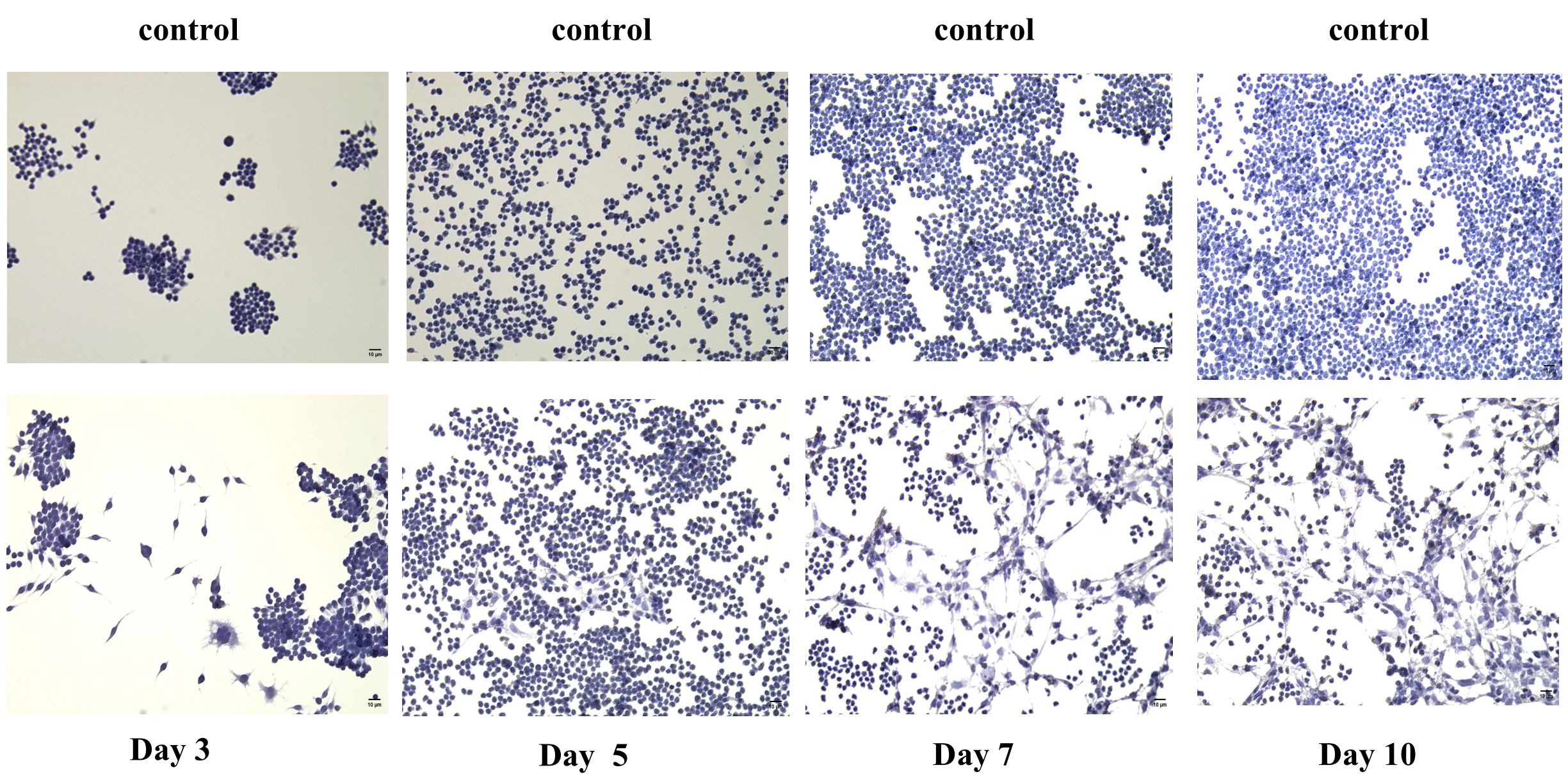
control
control
control
control
Day 3
Day 5
Day 7
Day 10

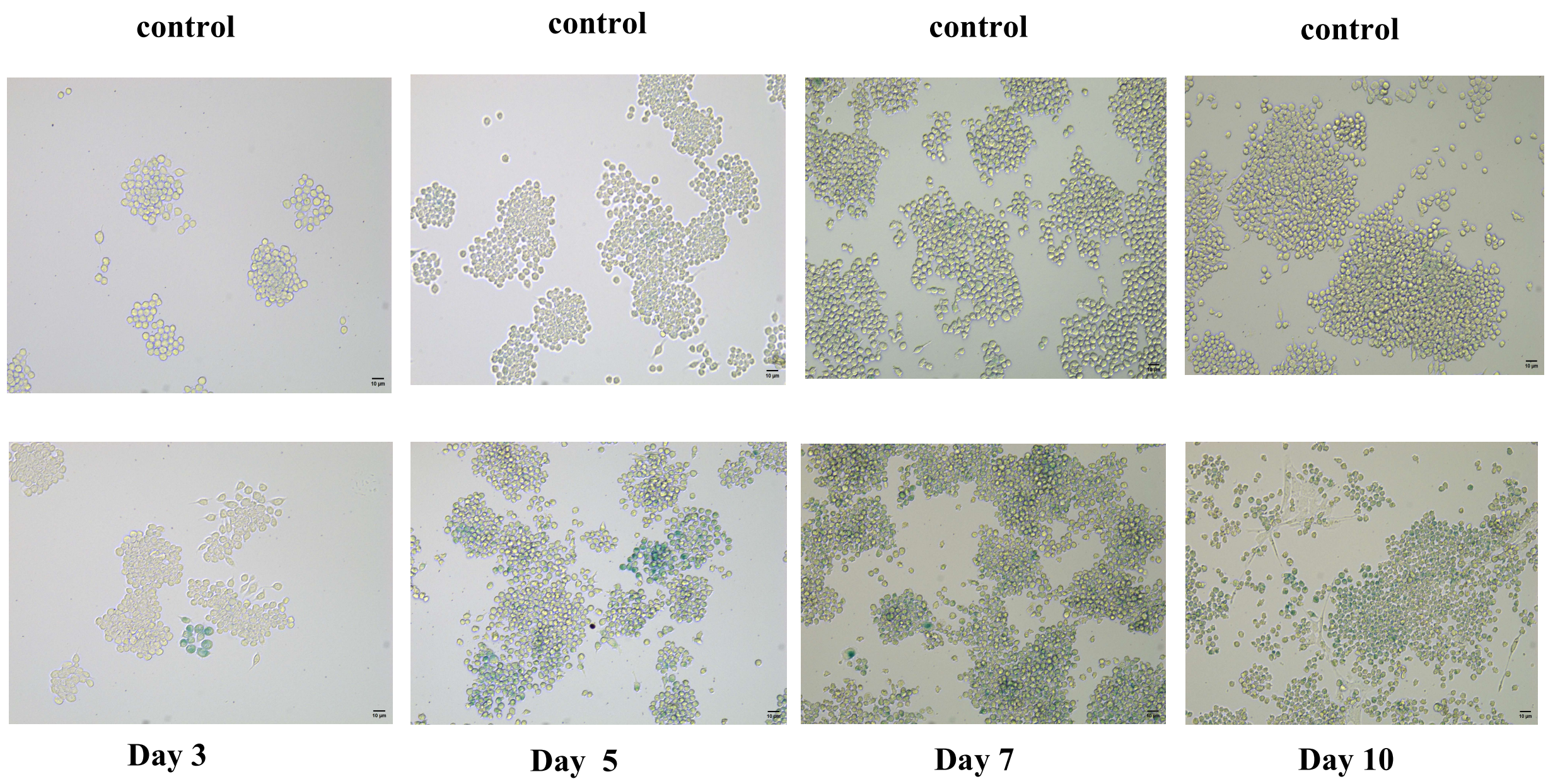
control
control
control
control
Day 3
Day 5
Day 7
Day 10

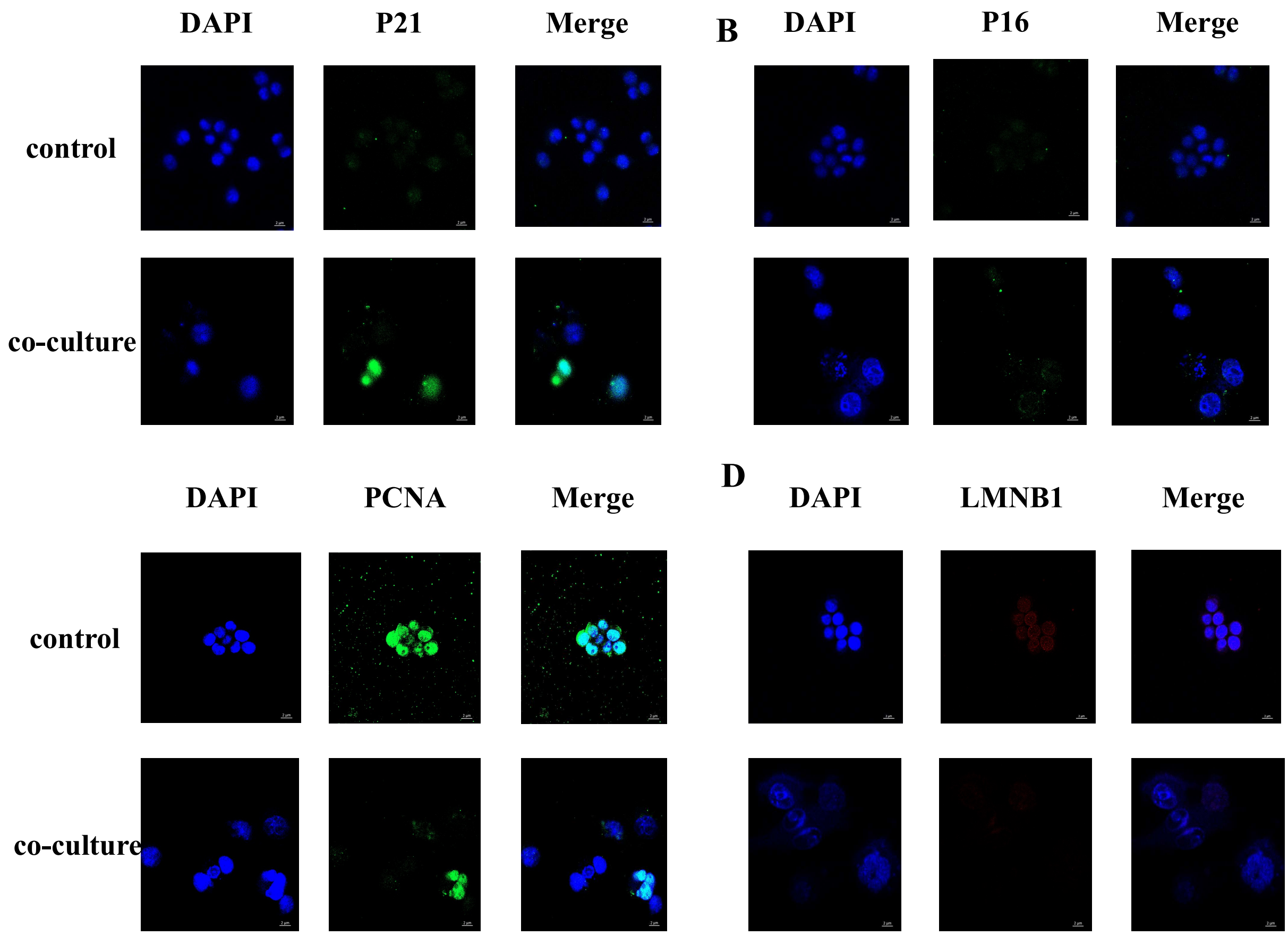
DAPI
P21
Merge
B
DAPI
P16
Merge
control
co-culture
D
DAPI
PCNA
Merge
DAPI
LMNB1
Merge
control
co-culture

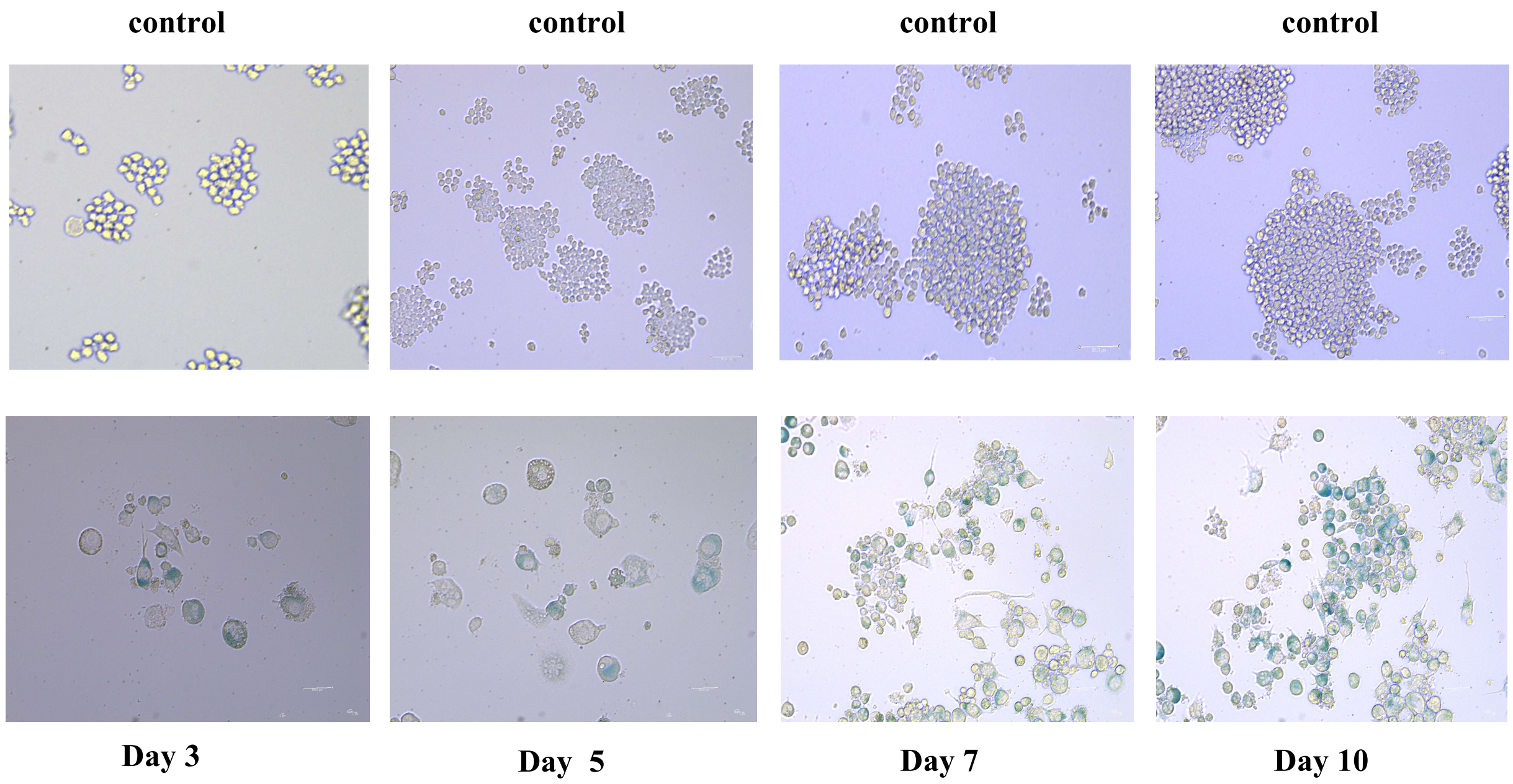
control
control
control
control
Day 3
Day 5
Day 7
Day 10

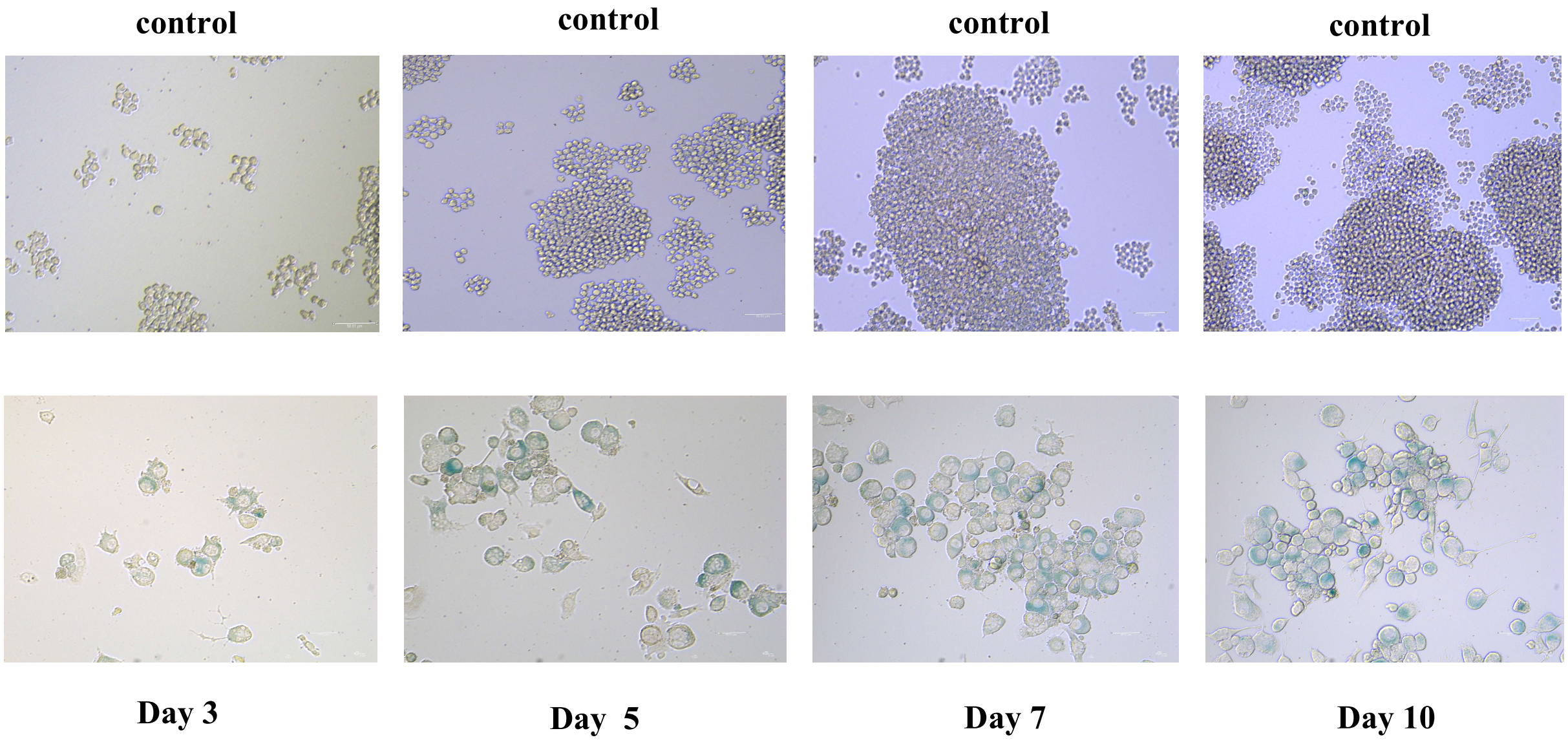
control
control
control
control
Day 3
Day 5
Day 7
Day 10

F

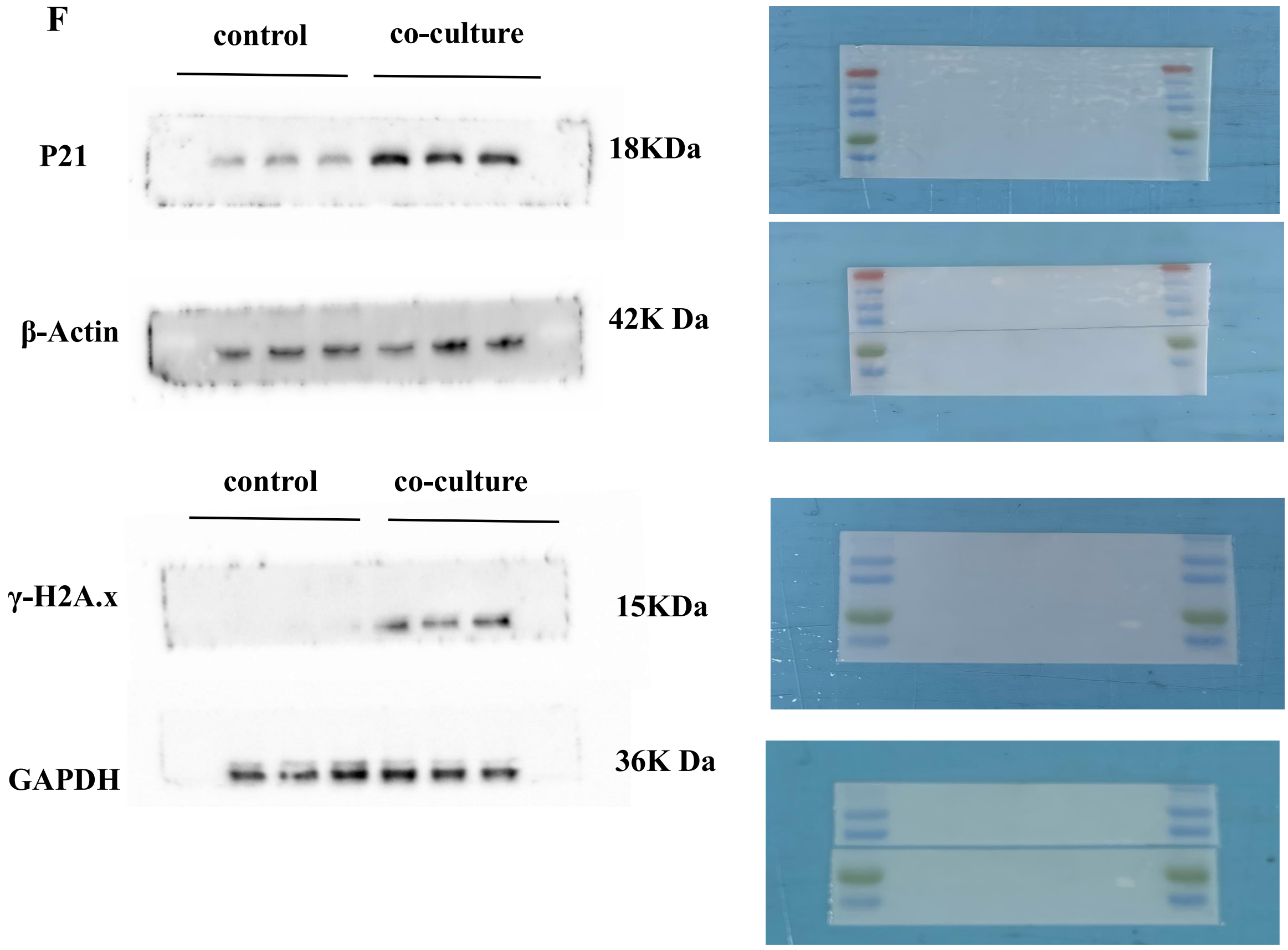
control
co-culture
P21
18KDa
β-Actin
42K Da
control
co-culture
γ-H2A.x
15KDa
GAPDH
36K Da

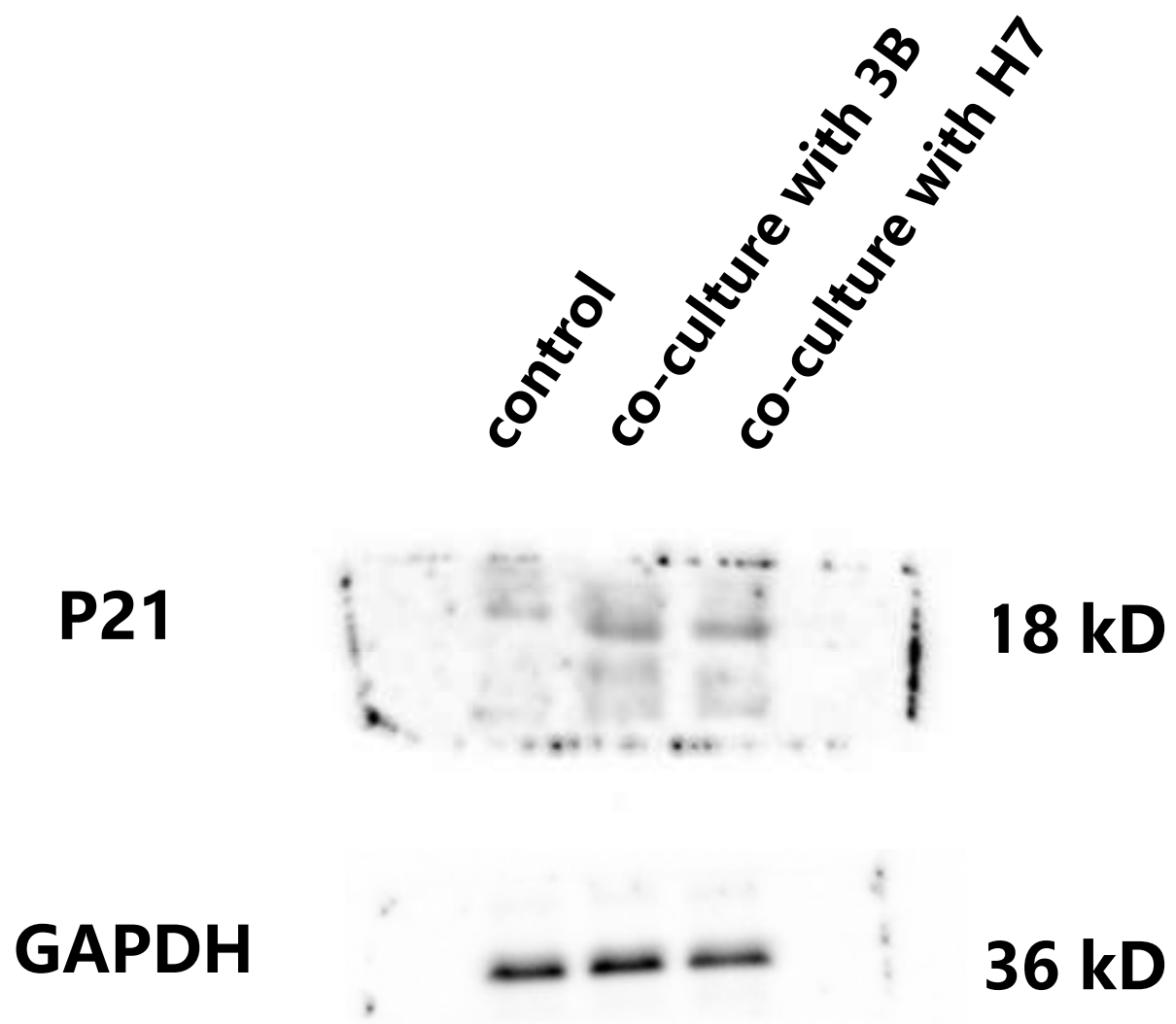
control
co-culture with 3B
co-culture with H7
P21
18 kD
GAPDH
36 kD

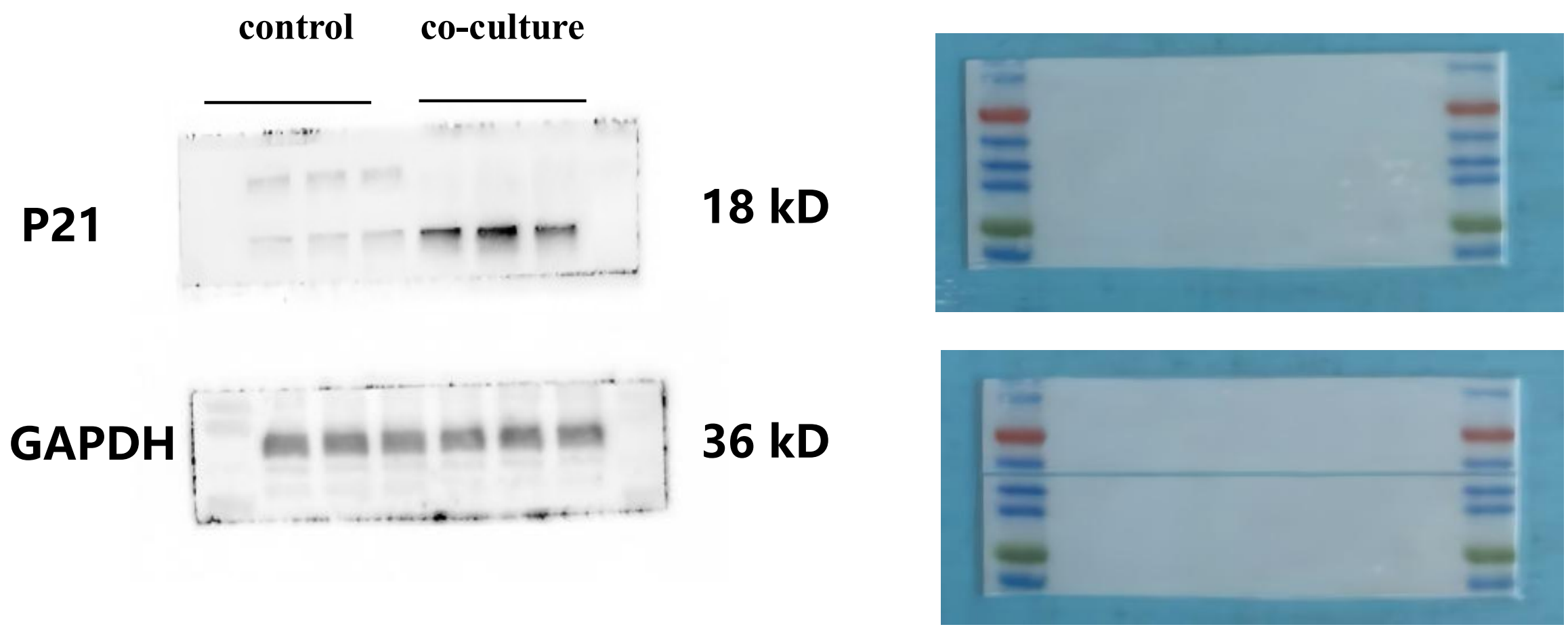
control
co-culture
P21
18 kD
GAPDH
36 kD
