# Supplementary material for: Induction of senescence like macrophages by hepatocellular carcinoma cells in vitro
Source: Discov Oncol. 2026 Jun 1;17:1117. doi: 10.1007/s12672-026-05348-8 (PMC13437835; doi:10.1007/s12672-026-05348-8)
Supplement: Supplementary file 2 [file 12672_2026_5348_MOESM1_ESM.docx]

Figure S1. A）Sa-β-Gal staining, respectively in two group on day 3,5,7,10 cultured at a density of 1 × 103 cells per well. B）Sa-β-Gal staining, respectively in two group on day 3,5,7,10 cultured at a density of 3 × 103 cells per well C) The quantification of Sa-β-Gal staining cell number in co-culture group on day 3,5,7,10 cultured at a density of 1 × 103 cells. D）The quantification of Sa-β-Gal staining cell number in co-culture group on day 3,5,7,10 cultured at a density of 3 × 103 cells. Signiﬁcance is noted as * p < 0.05, ** p < 0.01, and *** p < 0.001, **** P < 0.0001, ns: no significance.

Figure S2: A, B) Western blotting was utilized to evaluate protein expression levels of p21 in THP1 of control and co-culture group. C, D) Western blotting was utilized to evaluate protein expression levels of p21 in BMDM of control and co-culture group. E) Image of macrophages with internalized beads in BMDM of control group and co-culture group, F) and the results of the quantitative analysis are shown on the right. G) The expression of MHC Class II was assessed by flow cytometry in BMDM of control group and co-culture group, D) and the results of the quantitative analysis are shown on the right.
